# Comparison of the Cobas 4800 HPV and HPV 9G DNA Chip Tests for Detection of High-Risk Human Papillomavirus in Cervical Specimens of Women with Consecutive Positive HPV Tests But Negative Pap Smears

**DOI:** 10.1371/journal.pone.0140336

**Published:** 2015-10-15

**Authors:** Sun-Young Jun, Eun Su Park, Jiyoung Kim, Jun Kang, Jae Jun Lee, Yoonjin Bae, Sang-Il Kim, Lee-So Maeng

**Affiliations:** Department of Pathology, Incheon St. Mary’s Hospital, Incheon, The Catholic University of Korea, Incheon, Republic of Korea; Rudjer Boskovic Institute, CROATIA

## Abstract

Detecting high-risk (HR) HPV is important for clinical management of women with persistent HPV-positive and Pap-negative results. The Cobas 4800 HPV test is the first FDA-approved HPV DNA test that can be used alone as a first-line screening tool. The HPV 9G DNA chip test is a PCR-based DNA microarray assay. We evaluated the patients of consecutive HPV-positivity on HPV 9G DNA chip test without cytologic abnormalities. We then compared the performances of HPV 9G DNA chip and the Cobas 4800 HPV tests for detecting HR HPV with each other and confirmed HPV genotyping using direct sequencing. All 214 liquid-based cytology specimens were collected from 100 women with consecutive HPV-positive and Pap-negative results on the HPV 9G DNA chip test between May 2012 and Dec 2013, but only 180 specimens were available for comparing HPV test results. The HPV 9G DNA chip and the Cobas 4800 HPV tests agreed with each other in 81.7% of the samples, and the concordance rate was greater than 97.2% for detecting HPV-16 or -18. For HR genotypes other than HPV types 16 and 18, the two tests agreed for 81.1% of the samples. The sensitivity of both assays for detecting HR HPV was 100%, regardless of HR genotypes. The HPV 9G DNA chip test may be as effective as the Cobas 4800 HPV test in detecting HR HPV, and has a similar ability to identify HPV-16 and -18.

## Introduction

The Papanicolaou (Pap) screening test has significantly decreased the incidence of cervical cancer and its associated mortality during the last century. However, the Pap smear has low sensitivity and low reproducibility, in particular, for detecting high-grade squamous intraepithelial lesions (HSIL) [1]. Therefore, human papillomavirus (HPV) DNA co-testing with the Pap smear is recommended in order to improve the detection of precancerous cervical lesions. Adjunctive HPV testing increases the sensitivity of screening for HSIL and allows for extension of the screening interval [1]. Furthermore, in April 2014, the FDA approved the use of the Cobas 4800 HPV test (Roche Molecular Systems Inc., Pleasanton, CA, USA) alone as a first-line screening tool in women 25 years and older. The Cobas 4800 HPV test, firstly approved by the FDA in 2011, is a novel molecular method based on real-time PCR. It can identify HPV-16, HPV-18, and 12 other high-risk (HR) HPVs (HPV-31,-33,-35,-39, -45, -51, -52, -56, -58, -59, -66 and -68, as a pooled result) [2].

HPV infection, which is detected in 80% of SIL lesions, is the most common risk factor for cervical cancer [3]. HPV genotypes are commonly categorized as high (HR) and low-risk (LR) types, depending on their oncogenic potential. At least 18 HR HPV genotypes have been identified in human genital tracts; the most prevalent ones worldwide are HPV-16 and HPV-18 [1]. The estimated 12-month risk of developing HSIL in HPV-positive and Pap-negative women ranges from 0.8–4.1% [4]. Current guidelines for clinical management of HPV-positive and Pap-negative women recommend 1) repeated co-testing in 12 months, or 2) immediate specific genotyping for HPV-16 and/or HPV-18 [4]. If HPV-16 or -18 is detected in a woman who tests HPV-positive but Pap-negative, colposcopic examination is recommended.

In East Asia including Korea, the distribution of HPV genotypes is significantly different from that of Western populations. Specifically, HPV-58 is rare worldwide but is commonly found in East Asia [4–7]. HPV-58 ranks third in Asia overall, but contributes to only 3.3% of cervical cancers globally [7]. Interestingly, the 5-year cumulative incidence rates of HSIL in Korean women who are HPV-positive and Pap-negative were higher in HPV-58 positive cases (34.0%) than in HPV-16 positive cases (28.0%) and in other types of HR-HPV positive cases (5.5%) [4]. The identification of the HPV-58 genotype is important for the management of women who are HPV-positive and Pap-negative in a country with a high prevalence of HPV-58 infection [4]. Accordingly, HPV genotype-specific testing for HPV-16 and/or HPV-18 in HPV-positive and Pap-negative women may be an ineffective screening strategy, especially in Korea [4].

Several HPV DNA genotyping tests (HPV DNA chips) are commercially available in Korea and have been widely used along with the Hybrid Capture assay because of their equivalent sensitivity and specificity for the detection of HSIL, as well as the concordance among various HPV DNA tests [8, 9]. The HPV DNA chip is a PCR-based DNA microarray system that enables rapid, easy, and convenient genotyping of HR and LR HPV types at the same time. The detection of individual HR HPV types may provide more information than other HPV DNA tests. The HPV 9G DNA chip (Biometrix Technology Inc., Chuncheon, South Korea) is a commercial HPV DNA chip that detects 19 HPVs, including14 HR types identical to those detected by the Cobas 4800 HPV test and 5 LR types (HPV-6, -11, -34, -40, and -42) [9, 10]. The HPV 9G DNA chip provides 100% sensitivity and specificity for detection of 19 HPV genotypes in clinical samples [9]. However, even though the HPV DNA genome is detected on gel electrophoresis for PCR products of clinical samples, specific spots for HPV genotypes may be absent on the HPV DNA chip. Those clinical samples are designated as ‘HPV-other type’. When women consecutively test ‘HPV-other type’ without cytologic abnormalities, the significance of this result is unclear and it is consequently difficult to manage these women in clinical practice.

In this study, we evaluated patients with consecutive HPV positivity without cytologic abnormalities using the HPV 9G DNA chip test and investigated the distribution and change in HPV genotypes. In addition, we compared the performances of the HPV 9G DNA chip and the Cobas 4800 HPV tests for detecting HR HPV DNA with each other and confirmed HPV genotyping using direct sequencing.

## Materials and Methods

### Patients and samples

This study was approved by the Institutional Review Board of the Catholic University of Korea, Incheon St. Mary’s Hospital (No. OC14EISI0081). No consent was given because data were analyzed anonymously. In total, 2023 women who were co-tested using liquid-based cytology and HPV 9G DNA chip test between May 2012 and December 2013 at Incheon St. Mary’s Hospital were selected after searching our electronic pathologic database. Of these 2023 women who underwent co-testing, 323 (16.0%) were determined to be cytology-negative but HPV-positive on the initial screening test without previous cytologic/histologic abnormalities; HR HPV (128/2023 patients, 6.3%), LR HPV (24/2023, 1.2%), and HPV-other type (171/2023, 8.5%). Among these 323 patients, 100 had two or more consecutive HPV-positive and Pap-negative results by successive HPV co-testing. Of these 100 patients, 14 patients had three consecutive HPV-positive and Pap-negative results. Finally, we collected all 214 cervical swab specimens from these 100 women who had consecutive HPV-positive and Pap-negative results for the study. All women did not undergo gynecologic procedures such as colposcopic biopsy, conization, or hysterectomy. All specimens were placed in a ThinPrep PreservCyt solution (Hologic Inc., Malborough, MA, USA), and then stored at -70°C until they were assayed for HPV tests. The HPV 9G DNA chip and the Cobas 4800 HPV tests were performed by using the specimens from the same vials.

### HPV genotyping by HPV 9G DNA chip test

HPV 9G DNA chip tests were performed and interpreted according to the manufacturer’s protocol [9, 10]. The whole HPV genomic DNA extracted from cervical swab samples was amplified by duplex PCR to generate amplicons. Five microliters of Cy5-labeled PCR product was subjected to agarose gel electrophoresis using 2% agarose standard run in 1X Tris-borate EDTA. Five microliters of this PCR product was used for further hybridization experiments for HPV detection and specific genotyping. HPV amplicons could be hybridized with type-specific oligonucleotide probes and visualized on HPV 9G DNA chips as double-positive spots when HPV DNA was present in the amplified PCR product. However, as mentioned earlier, samples that showed a positive band for the HPV DNA genome on gel electrophoresis but were absent on the spots for 19 HPV genotypes on the HPV 9G DNA chip were designated HPV-other type, as they can be visualized on the spots for the positive control [9]. None of the negative controls (without DNA) revealed HPV positivity.

### High-risk HPV detection by the Cobas 4800 HPV test

HR HPV detection for the Cobas 4800 HPV test was also performed in all cervical swab specimens using the manufacturer’s protocol [11]. The Cobas 4800 HPV test utilizes a mixture of multiple primers and probes for amplifying and detecting human beta-globin gene from cervical cells as a target for internal quality control, as well as HPV DNA from cervical swab samples [11]. The Cobas 4800 HPV test is carried out using the Cobas 4800 system and the results are regarded as positive when the *C*_*T*_ from a sample is less than 40.0.

### HPV genotyping by direct sequencing

For the samples with discrepant results between two HPV detection assays, their HPV genotypes were identified using PCR and sequencing. PCR was performed using the general primer MY09/11 [12] and type specific primers (S1 Table). We designed type specific primers for identification of multiple HPV infection. After PCR amplification, the products were electrophoresed and the DNA of samples with a positive band was purified. Sequencing PCR was performed using MY09/11 and type specific primers, and the respective sequences of the HPV DNA regions corresponding to two primer sets were read using the Applied Biosystems (ABI) 3730XL DNA analyzer (Life Technologies Co., Carlsbad, CA, USA). Resulting DNA sequences were analyzed using the Basic Local Alignment Search Tool (BLAST) database on the website of the NCBI to confirm specific genotypes.

### Statistical analysis

Statistical analyses were performed using Analyse—it Method Evaluation Edition, version 2.22, software (Analyse-it Software Ltd., Leeds, UK). The agreement rate, kappa coefficient (*k*) with 95% confidence intervals (CIs), proportion of positive agreement (P*pos*), proportion of negative agreement (P*neg*), and McNemar’s *p* value were calculated to estimate the concordance between the results of two different tests. The *k* result was interpreted as follows: values < 0 as indicating no agreement and 0–0.20 as slight, 0.21–0.40 as fair, 0.41–0.60 as moderate, 0.61–0.80 as substantial, and 0.81–1 as almost perfect agreement. P*pos* was calculated as twice the number of agreed positives/(total number of specimens + number of agreed positives—number of agreed negatives), while P*neg* was calculated as twice the number of agreed negatives/(total number of specimens—number of agreed positives + number of agreed negatives). *P* values < 0.05 denoted statistical significance. Sensitivities, specificities, and 95% CIs of the Cobas 4800 HPV and the HPV 9G DNA chip tests were calculated based on the diagnostic accuracy criteria obtained from the genotyping results of the two HPV DNA tests and direct sequencing. When the results of two tests were concordant, they were regarded as true positive or negative. When there were discrepancies among the results, the diagnostic accuracy criteria were determined from the results of the HPV genotyping using direct sequencing.

## Results

### Characteristics of the study population

All 100 women with a median age of 49 years (range: 23–73 years) who were HPV-positive but Pap-negative at baseline were included in the analysis. Six women were under 30 years old. The follow-up interval between HPV co-testing ranged from 1.0 to 16.0 months (mean, 6.2 months; median, 6.0 months). Most women (*n* = 90) received short-term follow-up examinations for < 1 year.

At their initial HPV test, HR HPV infection was detected in 36 and LR HPV infection was detected in 13 women, while 51 women tested as HPV-other type. A single HPV type was detected in 96 women and ≥ 2 HPV types were detected in 4 women at their initial HPV test. During the follow-up period, consecutive HR HPV infection was detected in 17 women. Forty-one women showed various changes in HPV genotyping results; 32 women changed from HR HPV to LR HPV or HPV-other type, and 9 from LR HPV to HPV-other type. Thirty eight women consecutively tested positive for HPV-other type infection. Consecutive LR HPV infection was detected in only 4 women.

### HPV genotyping by HPV 9G DNA chip test

All 214 cervical swab specimens of 100 women were tested with the HPV 9G DNA chips. Among 214 cervical swab specimens, HR HPVs were identified in 72 samples (33.6%). Of these 72 HR HPV-positive samples, 6 were co-infected with LR HPV.LR HPVs alone were seen in 20 samples (9.3%). HPV-other type was found in 122 samples (57.0%). HPV-16 was the most common genotype of HR HPV (19/72, 26.4%), either alone or in combination with other types. HPV-18 was observed alone in 7 samples. HPV-58 was the second most common genotype of HR HPV (10/72, 13.9%). All of HPV-58 presented as single infection, except for the one exhibiting co-infection with HPV-16. The remaining types of HR HPV included HPV-68 (7/72, 9.7%), HPV-66 (6/72, 8.3%), HPV-33 (5/72, 6.9%), HPV-52 (5/72, 6.9%), HPV-39 (5/72, 6.9%), HPV-51 (4/72, 5.6%), and HPV-56 (4/72, 5.6%). HPV-31, -35, -45, and -53 were infrequently observed (less than 5%).

### Concordance among the results for the Cobas 4800 HPV and HPV 9G DNA chip tests

Only 180 out of 214 cervical swab specimens were available for comparison of HPV test results. Thirty four samples were not successfully analyzed by the Cobas 4800 HPV test due to a lack of remnant DNA or its degradation.

The results of the Cobas 4800 HPV and HPV 9G DNA chip tests based on the detected genotypes of HR HPVs are summarized in Table 1. In all of the 180 samples, overall positive rates for HR HPV were 38.3% (69/180) on the Cobas 4800 HPV test and 31.1% (56/180) by the HPV 9G DNA chip test, regardless of HPV genotypes. HPV-16 and HPV-18 were detected in more samples by the HPV 9G DNA chip test, though the positive rates of HR genotypes other than HPV types 16 and 18 were higher on the Cobas 4800 HPV test.

**Table 1:** Concordance of results between the Cobas 4800 HPV and the HPV 9G DNA chip tests according to HR HPV genotypes.

HR HPV genotype (*n* = 180)	Cobas 4800 HPV result	HPV 9G DNA chip result (*n*)		Total (*n*)	Agreement rate (%)	*k* (95% CI)	P*pos*	P*neg*	*P* value^a^
		**+**	**-**						
**Any of 14 HR HPVs**	**+**	46	23^b^	69	81.7%	0.598 (0.474–0.722)	0.736	0.860	*0*.*035*
	**-**	10^c^	101	111					
	**total**	56	124	180					
**16**	**+**	10	1^d^	11	97.2%	0.785 (0.600–0.971)	0.800	0.985	*0*.*375*
	**-**	4^e^	165	169					
	**total**	14	166	180					
**18**	**+**	3	0	3	99.4%	0.854 (0.570–1.000)	0.857	0.997	*1*.*000*
	**-**	1^e^	176	177					
	**total**	4	176	180					
**Other than 16 and 18**	**+**	33	26^f^	59	81.1%	0.535 (0.394–0.676)	0.660	0.869	*0*.*003*
	**-**	8^g^	113	121					
	**total**	41	139	180					

Abbreviations: HR HPV, high-risk human papillomavirus; *k*, kappa coefficient; CI, confidence interval; P*pos*, proportion of positive agreement; P*neg*, proportion of negative agreement.

^a^Statistically significant by McNemar’s test (*p*< 0.05).

^b^Sequencing results for detecting HR HPV (*n*): negative (11), could not be tested (12)

^c^Sequencing results for detecting HR HPV (*n*): negative (3), could not be tested (7)

^d^Sequencing results for detecting HR HPV (*n*): negative (1)

^e^Sequencing results for detecting HR HPV (*n*): could not be tested

^f^Sequencing results for detecting HR HPV (*n*): negative (12), could not be tested (14)

^g^Sequencing results for detecting HR HPV (*n*): negative (3), could not be tested (5)

**Fig 1:**
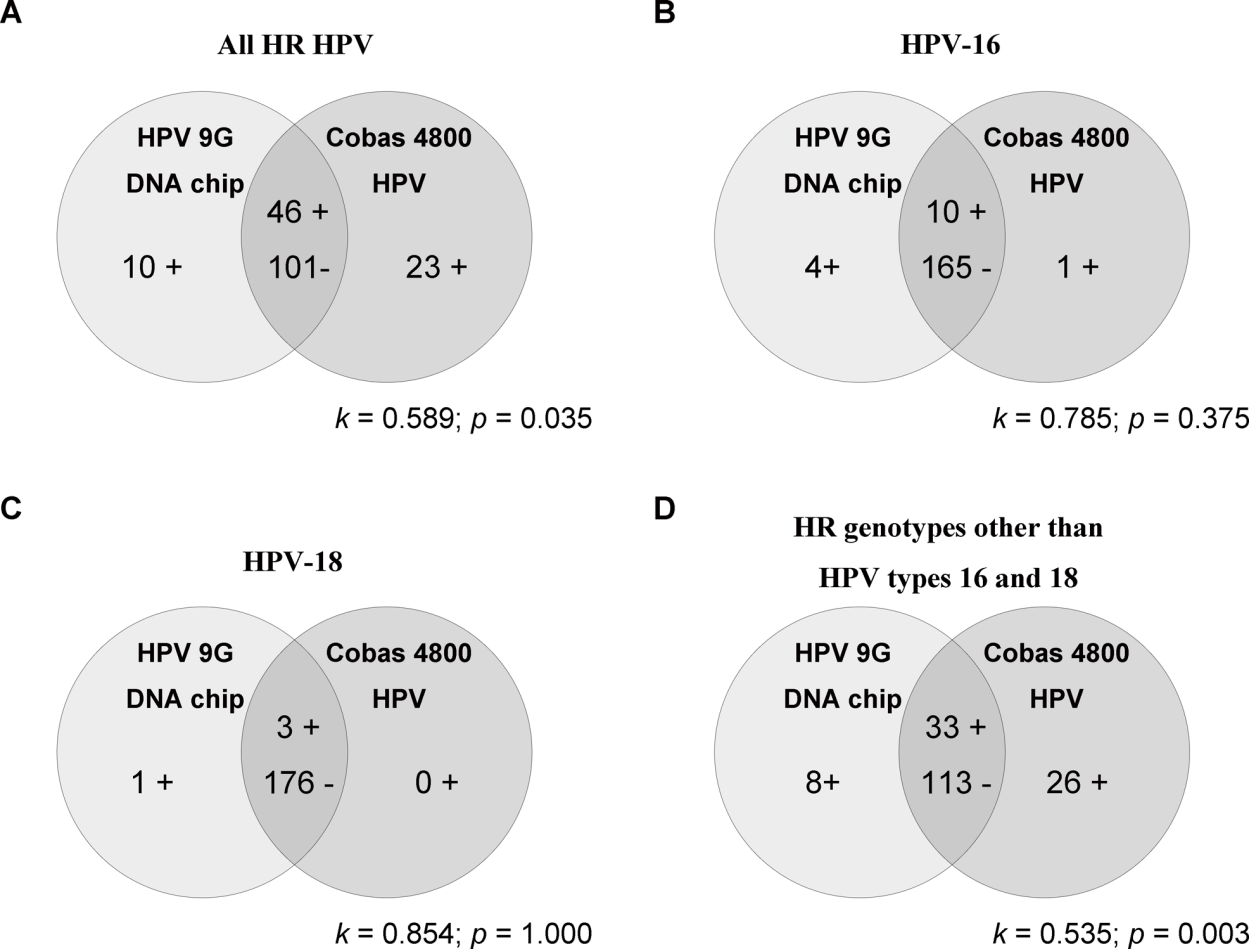
Concordance between the results of two HPV DNA tests according to high-risk (HR) HPV genotypes. (Abbreviations: *k*, kappa coefficient).

Regardless of HR HPV genotypes, the results of the two tests moderately agreed in 81.7% of all cases (*k* = 0.598) when detecting HR HPV (Fig 1). The proportions of positive and negative agreement for HPV status of any of the 14 HR HPV types were 0.736 and 0.860, respectively. The proportion of positive agreement was lower than that of negative one, ranging from 0.660 in HR HPV-other than types 16 and 18 to 0.857 in HPV-18. Among the 23 samples with positive HR HPV results only on the Cobas 4800 HPV test, 11 were negative for HR genotypes by sequencing. On the other hand, 3 of 10 specimens which were positive for HR HPV only on the HPV 9G DNA chip test were negative for HR genotypes by sequencing. The other specimens with discrepant results were not able to be sequenced due to a lack of remnant DNA.

For the HPV-16 genotype, the results of the two assays corresponded well (concordance rate, 97.2%; *k* = 0.785; Fig 1). Four samples were positive for HPV-16 only on the HPV 9G DNA chip test, with the lack of remnant DNA making it difficult to identify HPV genotypes by sequencing. On the other hand, the one sample that tested positive for HPV-16 only on the Cobas 4800 HPV test was negative for HPV-16 by sequencing, suggesting false-positivity. The two assays produced almost perfect agreement for HPV-18 results with a concordance rate of 99.4% (*k* = 0.854; Fig 1). There was only one discrepant sample, which was positive for HPV-18 exclusively on the HPV 9G DNA chip test. Sequencing was impossible in this case due to a lack of DNA.

When considering the HR genotypes other than HPV types 16 and 18, the results of the two HPV detection assays moderately agreed with each other at a rate of 81.1% (*k* = 0.535), which was similar to the overall concordance rate between two assays regardless of HR HPV genotype (Fig 1). The Cobas 4800 HPV test produced 26 more positive results for the HR genotypes other than HPV types 16 and 18. Twelve of these specimens were available for sequencing and genotyping, all of which were observed negative for HR HPV genotypes. However, 8 were positive for HR genotypes other than HPV types 16 and 18 only on the HPV 9G DNA chip test. Three of them were available for sequencing, all of which were shown negative for HR HPV genotypes. Statistically significant differences in the detection of HPV in clinical samples were noted for HR HPV other than types 16 and 18 when considering overall positivity with any of the 14 HR HPV types (*p* < 0.05).

### HPV-other type and low-risk HPVs on HPV 9G DNA chip test

The HPV 9G DNA chip test produced 108 HPV-other type test results out of 180 specimens. Most (87/108, 80.6%) were negative for HR HPV genotypes, but the rest (21/108, 19.4%) showed different HR HPV genotypes on the Cobas 4800 HPV test. Among HR HPV-positive samples only on the Cobas 4800 HPV test, 20 were positive for HR genotypes other than HPV types 16 and 18; 11/20 samples were directly sequenced and no HR HPV was identified. One sample (1/108, 9.3%) was positive for HPV-16 on the Cobas 4800 HPV test, but no HR HPV was seen upon sequencing.

LR HPVs were identified in 16 out of 180 specimens by the HPV 9G DNA chip test. Fourteen specimens (14/16, 87.5%) were negative for HR HPV genotypes on the Cobas 4800 HPV test. Only two samples (12.5%) were positive for HR genotypes other than HPV types 16 and 18, but none could be sequenced for genotyping.

### Sensitivities and specificities of the HPV detection assays

The sensitivities and specificities of the Cobas 4800 HPV and the HPV 9G DNA chip tests according to the detected genotypes of HR HPVs are summarized in Table 2. Out of a total of 180 specimens, 147 showed concordant results of HR HPV in both assays, regardless of HPV genotypes; 46 samples were concordantly positive for HR HPV and regarded as true positive, whereas 101 were negative for HR HPV and regarded as true negative. Direct sequencing was performed in the discordant 33 specimens; 14 were negative for HR HPV and considered as true negative. Nineteen samples were not successfully tested for sequencing due to a lack of or degradation of DNA, so those were excluded from the evaluation of the sensitivities and specificities of the two HPV DNA tests. Both assays showed high sensitivity at 100.0% regardless of HR HPV genotypes. The HPV 9G DNA chip test showed higher specificity (97.4%) for the detection of HR HPVs than the Cobas 4800 HPV test (90.4%). For the HPV-16 genotype and the HR HPV genotypes other than HPV types 16 and 18, the HPV 9G DNA chip test showed higher specificities (100.0% and 97.6%, respectively) than the Cobas 4800 HPV test (99.3% and 90.4%, respectively). The specificities of both assays for detecting HPV-18 were 100.0%.

**Table 2:** Sensitivities and specificities of HPV detection tests for HR HPV genotypes.

Test	HR HPV genotype	Sensitivity (%)	95% CI (%)	Specificity (%)	95% CI (%)
**HPV 9G DNA chip**	**Any of 14 HR HPVs**	100.0	90.4–100.0	97.4	92.0–99.3
	**16**	100.0	65.5–100.0	100.0	96.9–100.0
	**18**	100.0	31.0–100.0	100.0	97.0–100.0
	**Other than 16 and 18**	100.0	87.0–100.0	97.6	92.6–99.4
**Cobas 4800 HPV**	**Any of 14 HR HPVs**	100.0	90.4–100.0	90.4	83.2–94.9
	**16**	100.0	65.5–100.0	99.3	95.8–100.0
	**18**	100.0	31.0–100.0	100.0	97.0–100.0
	**Other than 16 and 18**	100.0	87.0–100.0	90.4	83.5–94.7

Abbreviations: HR HPV, high-risk human papillomavirus; CI, confidence interval.

## Discussion

HPV-positive/Pap-negative is the most common positive screening result obtained from co-testing. In a previous report in the Western population, 3.6% of women aged over 30 years have this co-test result at baseline [13]. Song and colleagues [4] found that 11.6% of Korean women are HPV-positive but cytology-negative on the screening co-test. The reasons for differences in the rate between the two studies are uncertain. One possibility is that the two previous studies used different HPV genotyping methods. The former study used the Hybrid Capture 2, while the latter study used the HPV DNA chip. In Korea, several HPV DNA chips are commonly used as alternative and rapid means for detecting HPV in clinical samples. In general, amplification assays using PCR-based techniques, such as the HPV DNA chip, are more sensitive than liquid hybridization tests, such as Hybrid Capture 2 [14]. In our present study using the HPV 9G DNA chip, the rate of women with HPV-positivity without cytologic abnormalities (323 of 2023 patients; 16.0%) was similar to that of Song and colleagues [4]. Therefore, we think, higher percentage of women who are HPV-positive/Pap-negative in Korea might be related with the application of HPV DNA chip test. In addition, the HPV DNA chip yields confusing result in the form of undisclosed HPV type, such as HPV-other type, which occupies a large part of HPV-positive/Pap-negative results. In this present study, HPV-other type was frequently found in 52.6% (171/323) of women with HPV-positive/Pap-negative result. However, HR HPV genotype was not identified in sequenced samples showing HPV-other type in our study. Hence, we postulate that this unusual HPV genotyping result may be due to high sensitivity of the HPV DNA chip test, and may have little connection with HR HPV.

Several previous reports have demonstrated the usefulness of HPV DNA chips in comparison with Hybrid Capture 2 in the general population [14–16]. Diagnostic accuracies of HPV DNA chips including the sensitivity and specificity have been evaluated based on cytologic or histological abnormalities of the cervix. In the literature, HPV DNA chips are comparable to Hybrid Capture 2 with respect to their sensitivity and specificity for the detection of cervical intraepithelial neoplasia grade 2 or greater and their concordance with HPV positivity [8, 16]. The HPV 9G DNA chip has shown 100% sensitivity and specificity in the detection and discrimination of 19 HPV genotypes in clinical samples [9]. An et al. described two important characteristics that probably contribute to proficient genotyping by the HPV 9G DNA chip: 1) a high signal-to-background ratio for HPV 9G DNA chip of 200, compared to 2.5 to 5 for the other DNA chips, and 2) 100% target specificity of HPV 9G DNA chip (results that are 100% identical with those of sequencing analysis) [9]. Nevertheless, the HPV 9G DNA chip test has limited usage for HPV in East Asia.

Nowadays, other HPV detection assays utilizing the real-time PCR method have been newly developed. The Cobas 4800 HPV test was recently accepted by FDA as a primary cervical cancer screening tool alone. The potential benefits of using the HPV DNA test for primary cancer screening are increasingly apparent [17]. However, the high rate of insignificant HPV infections has been an impediment to its potential use, so improving the specificity of HPV DNA test is a crucial step for successful implementation of HPV-based cervical cancer screening [17]. No study has concurrently evaluated the Cobas 4800 HPV test and the HPV DNA chip assays for detecting HR HPV. To exclude patients with cervical cytologic abnormalities and to highlight its own ability of HPV DNA test for detecting HR HPV, we selected samples with consecutive Pap-negative and HPV-positive results for HPV DNA testing and directly compared the results of the HPV 9G DNA chip and the Cobas 4800 HPV tests. Moreover, we examined the ability of the two different HPV assays to identify HR HPV other than HPV types 16 and 18, especially HPV-58. Both assays can detect HPV-16, HPV-18, and 12 other HR HPVs; however, the Cobas 4800 HPV test cannot identify the 12 other HR genotypes individually but can only report a pool of 12 HR HPVs. There was a statistically significant difference in the detection of HR genotypes other than types 16 and 18 between two HPV DNA tests (*p*< 0.05). HPV-58 was the most frequently observed (*n* = 10) in the HR HPV genotypes other than HPV types 16 and 18, corresponding with previous studies [4]. The HPV 9G DNA chip test produced 2 more positive results for HPV-58 compared to the Cobas 4800 HPV test, but samples were not available for sequencing and genotyping. To better describe the utility of HPV DNA tests for the specific detection of HPV-58, further studies may be needed.

This study has a few limitations that should be discussed. First, most women in this study (*n* = 90) received short-term follow-up of HPV co-testing for < 1 year. Therefore, this might contribute to persistent HPV-positivity as well as various changes in HPV genotypes. Although the worldwide guidelines recommend retesting in 1 year for women with a baseline HPV-positive/Pap-negative screening results, such women return at varying intervals in practice [13]. In Korea, the retesting interval tends to be short because of general concern about high incidence of cervical cancer, easy access to co-testing, and low medical costs [8]. Accordingly, this study contributes to the understanding of the actual distribution and change in HPV genotypes in Korean patients with consecutive HPV positivity without cytologic abnormalities. Short-term intervals for HPV co-testing do not affect evaluation of the HPV 9G DNA chip and the Cobas 4800 HPV assays. Second, we could not perform direct sequencing and identify HPV genotypes for all samples showing discrepant results between two HPV detection assays due to a lack of or degradation of DNA. We could only evaluate the diagnostic accuracy of two HPV DNA tests in 161 out of 180 samples. Further studies may be needed to better define the comparative performance of HR HPV tests for screening purposes in a large number of patients, either in conjunction with cytology or as stand-alone tests.

In conclusion, we analyzed the results of the HPV 9G DNA chip test in the samples of consecutive HPV-positive/Pap-negative and compared its performance with that of the Cobas 4800 HPV test for detecting HR HPV by using sequencing and genotyping results as the reference standard. HPV-16 was the most common genotype and HPV-58 was the second most common type of HR HPV in the HPV-positive/Pap-negative samples. The overall concordance rate of the Cobas 4800 HPV and the HPV 9G DNA chip tests for detecting HR HPV was moderate at 81.7%. Two different HPV DNA tests had more substantial agreement for detecting HPV-16 and -18 and moderately agreed for the HR genotypes other than HPV types 16 and 18. Both assays exhibited 100% sensitivity for detecting HR HPV, while the HPV 9G DNA chip test showed higher specificity than the Cobas 4800 HPV test. This discrepancy arose mainly in the samples positive for HR HPVs other than HPV types 16 and 18. Hence, the HPV 9G DNA chip test may be as effective as the Cobas 4800 HPV test in detecting HR HPV, and has a similar advantage in identifying HPV-16 and -18 from clinical samples.

## Supporting Information

S1 Table Sequences of type specific primers. (DOCX)
