# Supplementary material for: Comparison of the Cobas 4800 HPV and HPV 9G DNA Chip Tests for Detection of High-Risk Human Papillomavirus in Cervical Specimens of Women with Consecutive Positive HPV Tests But Negative Pap Smears
Source: PLoS One. 2015 Oct 15;10(10):e0140336. doi: 10.1371/journal.pone.0140336 (PMC4607436; doi:10.1371/journal.pone.0140336)
Supplement: S1 Table — (DOCX) [file pone.0140336.s001.docx]

| Type | Accession No. | Primer | Sequence |
| --- | --- | --- | --- |
| HPV-11 | KC329894 | 11F | 5’-TGG AGT GCA CAG ACG GAG ACA TCA GAC AAC-3’ |
|  |  | 11R | 5’-TTA AAC AAT GCC TGT GCT TCC ACA GAA TTT-3’ |
| HPV-16 | KP874716 | 16F | 5’-TTT ATA CAT TAA AGG CTC TGG GTC TAC TGC-3’ |
|  |  | 16R | 5’-TAA GGT TTA TTG AAT ATT TGG GCA TCA GAG-3’ |
| HPV-33 | KF700164 | 33F | 5’-AAC TAT ACA CAA CAT TGA ACT ACA GTG CGT-3’ |
|  |  | 33R | 5’-ATC TAA AAC ATA TTC CTT TAA CGT TGG CTT-3’ |
| HPV-35 | JX129488 | 35F | 5’-AGA AGT GGA CAG ACA TTG TAA GGT GCG GTA T-3’ |
|  |  | 35R | 5’-GTC ATC TTC ATT TTC GTC CTC TAC ACT GGA-3’ |
| HPV-39 | KC470245 | 39F | 5’-AAG TAT GTA TGA CAG TTT CAT GTG TGA TTG-3’ |
|  |  | 39R | 5’-ACA AAA TGG CGA AGT ATA AAA TGT AGA AAC-3’ |
| HPV-42 | GQ472847 | 42F | 5’-CAG CTA AAC GTA AGA AAA CAC ACA AAT AGA-3’ |
|  |  | 42R | 5’-CTT ATT TTT CAA AGC CAG GAT TGT AGT TTA-3’ |
| HPV-51 | M62877 | 51F | 5’-TTC GGT TCG TGT ACT TTT AGT ATA TTT GCC-3’ |
|  |  | 51R | 5’-TTA AAT TAT TAT AGG GCG GAA AAC AGT GTG-3’ |
| HPV-56 | JX912947 | 56F | 5’-AGA AGC ACA GCT ATA ACA TGT CAA CGG GAA C-3’ |
|  |  | 56R | 5’-CTT ACA AAA CAA AAG CCA CAA TAA TGA CAC-3’ |
| HPV-58 | AB819279 | 58F | 5’-CAG ACT AAA ACG TTC GGC CCC TAC TAC CCG-3’ |
|  |  | 58R | 5’-GGA GGT AAA GTA AAA TGG AGGG CAG TAC TGT-3’ |
| HPV-66 | JN122292 | 66F | 5’-TTG ATT GTA AAC AAA CCC AGT TAT GTA TTG-3’ |
|  |  | 66R | 5’-GGG CAT CAT ATT TAG TTA ATG TGC TTT TAG-3’ |
